# Associations of Dietary Patterns and Dietary Index with Iron Deficiency Across Different Stages Among Children Aged 9–17 Years in Guangzhou, China: A Cross-Sectional Study

**DOI:** 10.3390/nu18101620

**Published:** 2026-05-20

**Authors:** Jie Huang, Jinhan Fu, Bingyu Liuzhang, Chunzi Zeng, Shiyun Luo, Yujie Peng, Yanyan Wang, Zhifeng Li, Yuting Qin, Wanzhen Zhong, Weiwei Zhang, Zhoubin Zhang, Longying Zha, Yan Li

**Affiliations:** 1 Guangzhou Center for Disease Control and Prevention (Guangzhou Health Supervision Institute), Guangzhou 510440, China; huangjie1026@126.com (J.H.); eggyeggsy@smu.edu.cn (B.L.); gzcdc_zengcz@gz.gov.cn (C.Z.); luoshy25@mail3.sysu.edu.cn (S.L.); 15103533398@163.com (Y.P.); gzcdc_wangyy@gz.gov.cn (Y.W.); jerrphen@icloud.com (Z.L.); gzcdc_qinyt@gz.gov.cn (Y.Q.); zhongwzh6@mail2.sysu.edu.cn (W.Z.); gzcdczhangww@foxmail.com (W.Z.);; 2 Institute of Public Health, Guangzhou Medical University & Guangzhou Center for Disease Control and Prevention (Guangzhou Health Supervision Institute), Guangzhou 510440, China; 3 Sanshui District Center for Disease Control and Prevention, Foshan 528199, China; fujh6@alumni.sysu.edu.cn; 4 School of Public Health, Southern Medical University, Guangzhou 510515, China; 5 School of Public Health, Sun Yat-Sen University, Guangzhou 510080, China

**Keywords:** dietary patterns, CDGI(2021)-C, iron deficiency, iron deficiency store, iron deficiency erythropoiesis, iron deficiency anemia, children

## Abstract

**Background**: Iron deficiency (ID) progresses through three stages: iron deficiency stores (IDS), iron deficiency erythropoiesis (IDE), and iron deficiency anemia (IDA). Neglecting subclinical ID may be harmful to school-aged children and increase the public health burden. Although diet is a key modifiable factor, most studies only focus on overall ID or merely the clinical IDA stage. This study combines a dietary index with pattern analysis to take advantage of their complementary strengths and explore their associations with ID progression. **Methods**: This cross-sectional study included 2493 participants from rural Guangzhou between June 2022 and May 2023. Demographic, lifestyle, anthropometric, and dietary data were collected via structured questionnaires. Blood samples were analyzed for iron status. Factor analysis identified dietary patterns, and the Chinese Dietary Guidelines Index for Children and Adolescents [CDGI(2021)-C] assessed dietary quality. We used ordinal logistic regression, multivariable logistic regression, and restricted cubic spline (RCS) models to examine dietary associations with ID stages. **Results**: IDS, IDE, and IDA proportions were 68.22%, 17.45%, and 14.33%, respectively. All four dietary patterns correlated positively with CDGI(2021)-C, most strongly for the fruit–vegetable (*r_s_* = 0.552) and cereal–tuber–legume patterns (*r_s_* = 0.386). Higher CDGI(2021)-C (OR = 0.852, 95% CI: 0.751–0.966, *p*-trend = 0.012), fruit–vegetable (OR = 0.866, 95%CI: 0.748–0.993, *p*-trend = 0.047), and meat–offal patterns (OR = 0.733, 95%CI: 0.611–0.868, *p*-trend < 0.001) were inversely associated with advancing ID stages, while the snack–fast food pattern was positively associated (OR = 1.233, 95% CI: 1.094–1.381, *p*-trend < 0.001). In IDS, higher adherence to CDGI(2021)-C, fruit–vegetable, and meat–offal patterns was associated with lower odds (all *p*-trend < 0.05). RCS showed nonlinear associations for the snack–fast food and cereal–tuber–legume patterns, with risk peaking at moderate-to-high adherence to these patterns (both *p*-nonlinear < 0.05). In IDE and IDA, the snack–fast food pattern risk rose steeply at moderate-to-high adherence (*p*-nonlinear = 0.036), whereas the cereal–tuber–legume pattern’s ORs fluctuated near 1 (*p*-nonlinear = 0.020). **Conclusions**: Dietary pattern and index analyses showed consistent associations across ID stages. Adherence to dietary guidelines slows ID progression, especially in early subclinical stages. More fruits, vegetables, and heme-iron-rich foods, alongside less fast food and snacks, slow ID progression. Though dietary intervention effects weaken in later stages, reducing fast food and snacks confers long-term benefits. These findings inform targeted nutrition policies to prevent ID progression in children.

## 1. Introduction

Iron is an essential micronutrient for hematopoiesis, oxygen transport, tissue respiration, and immune and central nervous system function [1]. Iron deficiency (ID) is a progressive disorder caused by insufficient dietary iron relative to physiological demands, or by excessive physiological and pathological losses [2]. It remains a major global public health challenge, with prevalence increasing from 0.98 billion in 1990 to 1.27 billion in 2021 and projected to reach 1.44 billion by 2050 [3]. Untreated ID may lead to irreversible brain damage, neurobehavioral disorders, immunosuppression, and stunted growth [4,5]. ID progresses through three sequential stages: iron deficiency stores (IDS), iron deficiency erythropoiesis (IDE), and iron deficiency anemia (IDA). Hemoglobin remains normal in IDS and IDE (subclinical ID), yet these stages are associated with impaired enzyme activity, neurotransmitter synthesis, abnormal behavior, and poor appetite [6].

Most current studies have focused on IDA in infants and preschool children, with limited attention to the early stages of ID in school-aged children [7,8]. Rapid pubertal growth greatly increases iron demand. Inadequate iron intake or low iron stores increase the risk of IDA. This underscores the importance of early ID detection and prevention. Socioeconomic status is a key determinant, with rural and impoverished children disproportionately affected [9,10]. In China, ID and IDA prevalence are markedly higher in rural children [11], making rural school-aged children a priority group for targeted prevention research.

Among the factors influencing iron homeostasis, diet is the most modifiable factor and plays a key role in preventing and managing ID progression [6]. Most previous studies have focused on iron supplementation [6,12,13] or single nutrients [14], with limited evidence on overall dietary patterns and food component interactions [15]. With advances in nutritional epidemiology, dietary pattern analysis has been widely used to examine diet–health associations. These methods are mainly classified as a priori or a posteriori approaches [16,17]. The a priori method uses dietary guidelines to establish standardized indices (e.g., Healthy Eating Index [HEI], Diet Quality Index [DQI]) for assessing dietary adherence and quality [18]. Higher dietary index scores are linked to better iron status and lower ID risk [19,20]. However, dietary indices are population specific. The Chinese Dietary Guidelines Index for Children (CDGI(2021)-C) is a validated tool for Chinese youth [21]. It has mainly been used in dietary quality and bone health studies [22], but its value in evaluating ID and its different stages remains unclear.

The a posteriori approach derives dietary patterns from dietary data using statistical methods (e.g., principal component analysis, factor analysis), objectively reflecting food preferences and eating habits. Current research has largely focused on associations between dietary patterns and overall ID or clinical manifestation (IDA). Few studies have examined the role of diet across the progressive stages of ID. For example, a Swedish study reported that a red meat-poultry pattern reduced ID risk, while a vegetarian–legume pattern increased risk [23]. An Indonesian study found no significant association between plant-based dietary patterns and IDA [24]. Our preliminary study showed that snack–fast food and cereal–tuber patterns may increase ID risk, whereas fruit–vegetable and meat–offal patterns were protective against ID [25].

Dietary indices and dietary pattern analysis have distinct dimensions and complementary advantages. Their combined use can better clarify the associations between diet and ID progression. Therefore, this study examined these dietary factors and the developmental changes in ID status among rural school-aged children aged 9–17 years in Guangzhou. We hypothesized that CDGI(2021)-C and dietary patterns exert consistent effects on the progression of ID, while displaying distinct magnitudes across its sequential developmental stages. This study provides evidence for early ID screening, targeted dietary interventions, and prevention strategies for rural children.

## 2. Materials and Methods

### 2.1. Study Population and Design

This cross-sectional study used data from the Guangzhou Student Nutrition and Health Surveillance (June 2022–May 2023), conducted by the Guangzhou Center for Disease Control and Prevention (GZCDC) among students aged 9–17 years. Participants were selected using a multi-stage stratified cluster-random sampling method. Twelve primary, junior, and senior high schools in rural areas were randomly chosen, followed by stratified sampling of five grades, with classes as the final sampling units. Detailed procedures have been described previously [25].

Sample size was calculated using the formula: $N=deff\frac{Z_{\alpha /2}^{2}\times p(1-p)}{\delta^{2}}$, δ=r×p [26]. N denotes the total sample size; $Z_{\alpha /2}$ is the critical value for a two-sided *α* in the standard normal distribution; P represents the target prevalence; *δ* is the margin of error, and deff is the design effect.

**Figure 1:**
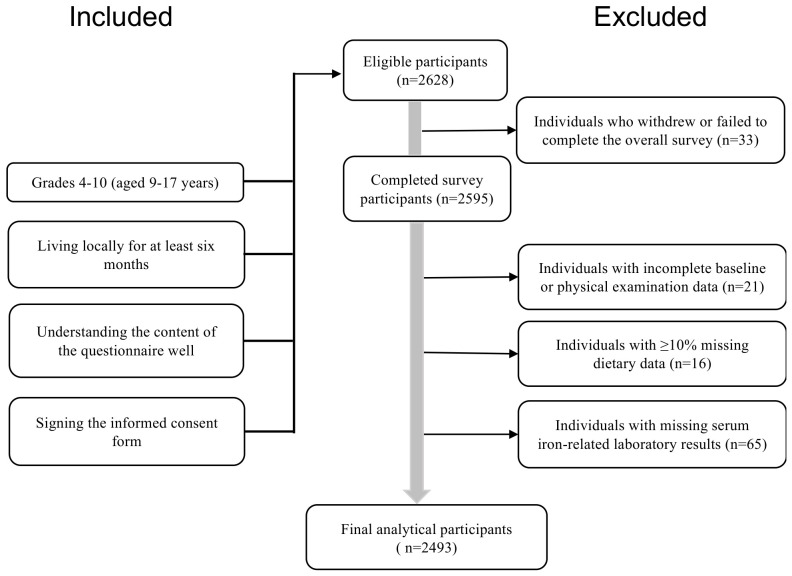
Selection process flowchart for participants.

Based on national and local data [27,28,29], ID prevalence (*p*) was conservatively estimated as 13%. With *α* = 0.05 ($Z_{\alpha /2}$= 1.96), *deff* = 2.0, relative error (*r*) = 20%, and 10% attrition, the minimum required sample size was 1429. A total of 2628 students met the eligibility criteria. Of these, 33 did not complete the full survey (due to withdrawal or missing data on dietary questionnaires, physical examinations, or blood collection), leaving 2595 participants with complete data. We further excluded 16 participants with ≥10% missing dietary data, 21 with incomplete baseline or physical examination information, and 65 with missing serum iron-related laboratory results. The final analytical sample consisted of 2493 participants (Figure 1).

This study was conducted in accordance with the Declaration of Helsinki and approved by the Ethics Committee of GZCDC (ethics number GZCDC-ECHR-2022P0036 and GZCDC-ECHR-2022P0038). Written informed consent was obtained from all participants and their legal guardians.

### 2.2. Data Collection

#### 2.2.1. Covariates

Demographic characteristics (age, sex, boarding status, and parental education) and lifestyle factors (smoking attempt, alcohol consumption, physical activity, and sleep duration) were collected through face-to-face interviews with children. All interviewers had received standardized training to ensure data quality.

Boarding status was classified as boarders or non-boarders. Parental education was categorized as primary school or below, middle school, high school, college degree or above, and unknown. A smoking attempt was defined as ever having smoked even one puff. Alcohol consumption was defined as ever having drunk a full cup of alcohol. Moderate-to-high-intensity physical activity was grouped as <3 days/week or ≥3 days/week. Sleep duration included nocturnal sleep and daytime napping. Height and weight were measured to the nearest 0.1 cm and 0.1 kg. Body mass index (BMI) was calculated as weight (kg)/height^2^ (m^2^). BMI z-scores were generated using WHO AnthroPlus 3.0 based on the 2007 WHO reference [30]. Nutritional status was classified as malnutrition (BMI z-score < −2), normal (−2 ≤ BMI z-score < 1), and overweight/obesity (BMI z-score > 1).

#### 2.2.2. Dietary Data

Children’s dietary intake over the past month was assessed using a 66-item semi-quantitative food frequency questionnaire (FFQ), including food consumption frequencies and portion weights. The FFQ was adapted from the 2015 China National Chronic Non-Communicable Disease and Nutrition Surveillance FFQ [31]. It was refined through a literature review and consultations with nutrition experts to better reflect local dietary habits. The reliability and validity of this FFQ have been reported previously [25]. Interviewers received standardized FFQ training from nutrition and food health professionals. They used food pictures and models to help participants identify food items and estimate portion sizes.

Overall diet quality was evaluated using a priori and a posteriori approaches. The Chinese Dietary Guidelines Index for Children and Adolescents (CDGI(2021)-C) was used as the a priori indicator. Scoring procedures have been described previously [21]. Owing to missing data on dark vegetables, salt, oil, and alcohol, CDGI(2021)-C was computed with 10 components, with tuber crops replacing other cereals and legumes (Supplementary Table S1). Total scores (0–75) were divided into quartiles (Q1–Q4).

Dietary patterns were derived using factor analysis. The 66 food items were grouped into 16 categories (Supplementary Table S2). Factor retention and factor loadings followed the criteria described in our previous study [25]. Participants were stratified into quartiles based on factor scores, with Q1 as the lowest and Q4 as the highest consumption.

### 2.3. Assessment of Iron Deficiency

Fasting venous blood was collected in serum separation and anticoagulant tubes. Serum was separated by centrifugation at 3000 rpm for 10 min at room temperature and stored at −80 °C until analysis. Serum ferritin (SF) was measured by latex-enhanced immunoturbidimetry, C-reactive protein (CRP) by immunoturbidimetry, serum iron (SI) and total iron-binding capacity (TIBC) by colorimetry, all on a Mindray BS-2000M automated analyzer from Mindray Corporation, Shenzhen, China. Hemoglobin (Hb) was determined in whole blood by the cyanmethemoglobin method. Transferrin saturation (TS) was calculated as (SI/TIBC) × 100%.

SF was used as the primary biomarker [32]. To adjust for inflammation, ID was defined by SF combined with CRP [6,33]. For participants aged ≥5 years, ID was defined as CRP ≤ 5 mg/L and SF < 25 μg/L, or CRP > 5 mg/L and SF < 32 μg/L.

SI and TS were used as auxiliary indicators. Potential ID was defined as SI < 9.0 μmol/L [6] or TS < 14% [34]. ID was staged into three groups according to the *Expert Consensus on the Prevention and Treatment of Iron Deficiency and Iron Deficiency Anemia in Children* [6]: iron deficiency stores (IDS): isolated SF deficiency; iron deficiency erythropoiesis (IDE): SF deficiency plus low SI and TS, without anemia; iron deficiency anemia (IDA): IDE plus anemia.

Anemia was defined using age- and sex-specific Hb cutoffs according to WHO 2011 criteria [35]: 5–11 years: <115 g/L; 12–14 years: <120 g/L; males ≥ 15 years: <130 g/L; non-pregnant females ≥ 15 years: <120 g/L.

### 2.4. Statistical Analysis

Questionnaire coding, processing, and validation were described previously [25]. Normality of continuous variables was tested by the Shapiro–Wilk test. Normally distributed data were presented as mean ± standard deviation (SD), non-normally distributed data as median (interquartile range, IQR). Continuous variables were compared using independent-sample *t*-tests, Mann–Whitney U tests, or Kruskal–Wallis tests. Categorical variables were reported as numbers (percentages) and analyzed using chi-square or Fisher’s exact tests.

We used ordinal logistic regression to assess dose–response trends across ordered ID stages: 0 = non-ID (reference), 1 = IDS, and 2 = IDE + IDA (groups combined owing to small sample sizes). Multivariable logistic regression examined associations between dietary indicators (lowest quartile as reference) and two binary outcomes: IDS vs. non-ID, and combined IDE + IDA vs. non-ID. We reported odds ratios (ORs) and 95% confidence intervals (CIs). Covariates were selected based on univariate analyses and literature. Three models were constructed as follows: Model 1 (crude), Model 2 (adjusted for age, sex, and BMI Z-score), and Model 3 (fully adjusted). Nonlinear associations were evaluated using restricted cubic spline (RCS) logistic regression with three knots.

All statistical analyses were performed using R 4.2.3. Two-sided *p*-values < 0.05 were considered statistically significant.

## 3. Results

### 3.1. Participant Characteristics

There were 1347 boys (54.03%) and 1146 girls (45.97%), aged 9–17 years, with a median age of 13.24 years (IQR: 11.26, 14.38). The median CDGI(2021)-C score was 43.75 (36.01, 51.18). The overall prevalence of ID was 12.88% (321/2493). Among the 321 children with ID, the proportions of IDS, IDE, and IDA were 68.22% (219/321), 17.45% (56/321), and 14.33% (46/321), respectively, corresponding to population prevalence rates of 8.78% (219/2493), 2.25% (56/2493), and 1.85% (46/2493), respectively.

The characteristics of participants stratified by ID status are shown in Table 1. Age increased gradually with the severity of ID, with the median age rising from 13.16 years in the non-ID group to 14.38 years in the IDA group (*p* < 0.001). Females and normal-weight children had a significantly higher prevalence of advancing ID stages (both *p* < 0.001). Boarding students had a higher prevalence of moderate-to-severe ID (*p* < 0.001). Median sleep duration decreased progressively from 9.09 h in the non-ID group to 8.25 h in the IDA group (*p* < 0.001). Individuals with lower exercise frequency had more advanced ID (*p* = 0.027). The CDGI(2021)-C score gradually declined as ID worsened (*p* < 0.001).

### 3.2. Dietary Patterns and Their Association with Dietary Index

#### 3.2.1. Dietary Patterns

**Figure 2:**
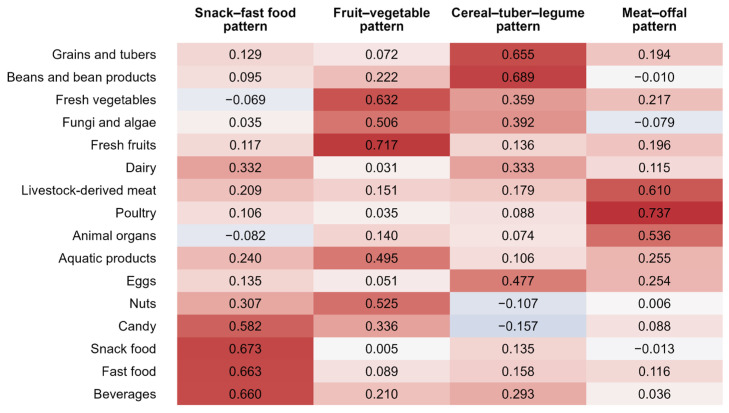
Heatmap of factor loadings for 16 food groups across dietary patterns identified by factor analysis. Red, white, and blue represent maximum, intermediate, and minimum factor loadings, respectively. Color intensity corresponds to the absolute value of the factor loadings.

Four major dietary patterns were extracted, explaining 45.48% of the total variance. A heatmap of factor loadings across the 16 food groups is presented in Figure 2. The snack–fast food pattern showed high loadings on processed items, including snacks, fast food, candy, and beverages. The fruit–vegetable pattern was characterized by high loadings on plant-based foods, including vegetables, fruits, fungi, and nuts. The cereal–tuber–legume pattern loaded on staple foods (grains, tubers, and legumes), along with eggs and dairy products. The meat–offal pattern loaded predominantly on animal-source foods such as meat, poultry, and offal. These four dietary patterns were adopted as core exposure variables in subsequent analyses.

#### 3.2.2. Distribution of Dietary Patterns and Dietary Index

Characteristics of participants in the lowest and highest quartiles (Q1, Q4) of each dietary pattern are shown in Supplementary Table S3. Each dietary pattern exhibited unique demographic and lifestyle profiles with significant differences between Q1 and Q4 across most examined variables (all *p* < 0.05). The distribution of all dietary patterns differed significantly across CDGI(2021)-C quartiles (all *p* < 0.05). Higher CDGI(2021)-C scores were consistently associated with higher quartiles of each dietary pattern, with the strongest associations observed for the fruit–vegetable pattern (Q4 vs. Q1: 91.75% vs. 8.25%, *p* < 0.001) and cereal–tuber–legume pattern (Q4 vs. Q1: 75.67% vs. 24.33%, *p* < 0.001).

Characteristics of participants in the lowest and highest quartiles (Q1, Q4) of CDGI(2021)-C scores are presented in Supplementary Table S4. Compared with participants in Q1, those in the highest CDGI(2021)-C quartile (Q4) were younger, more likely to be male, non-boarders, and had higher parental educational levels (all *p* < 0.05). For lifestyle behaviors, individuals with higher CDGI(2021)-C scores engaged in more frequent moderate-to-high-intensity physical activity and had longer sleep duration (all *p* < 0.001).

#### 3.2.3. Association Between Dietary Patterns and CDGI(2021)-C

Spearman correlation analysis was performed to explore the associations between dietary pattern factor scores and CDGI(2021)-C scores (Table 2). All four patterns were positively correlated with CDGI(2021)-C scores, with Spearman correlation coefficients ranked as follows: fruit–vegetable pattern (r_s_ = 0.552, *p* < 0.001), cereal–tuber–legume pattern (r_s_ = 0.386, *p* < 0.001), snack–fast food pattern (r_s_ = 0.168, *p* < 0.001), and meat–offal pattern (r_s_ = 0.068, *p* = 0.001).

### 3.3. Associations of Dietary Patterns and Dietary Index with Ordered Stages of Iron Deficiency

**Figure 3:**
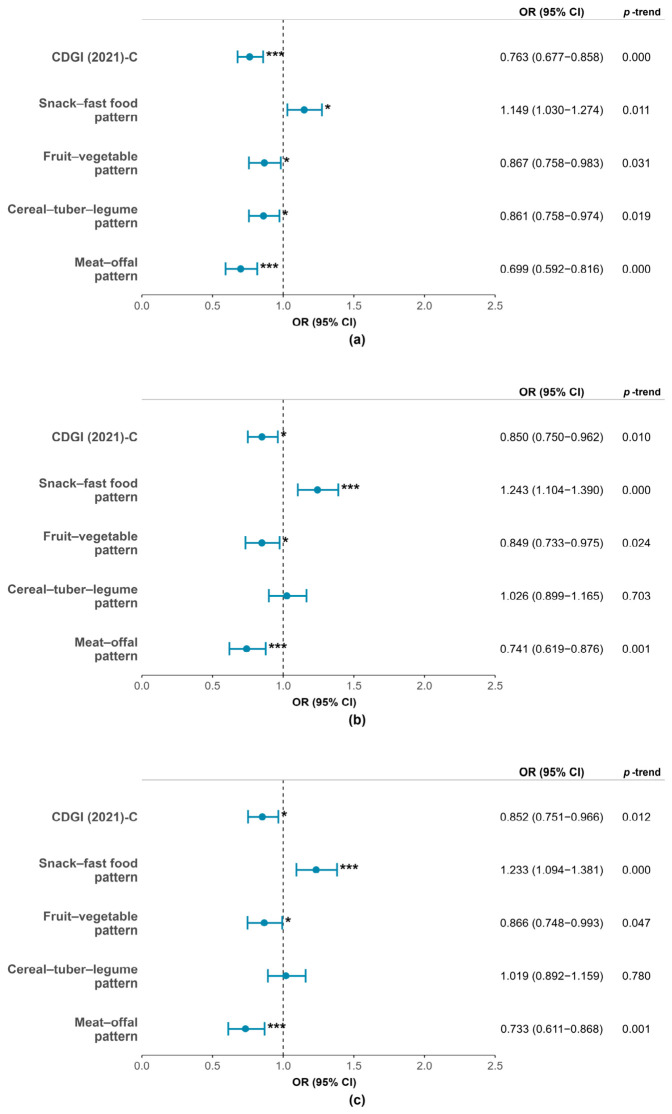
Ordinal logistic regression was performed to present the odds ratios (ORs) and 95% confidence intervals (95% CIs) for the associations of dietary pattern scores and CDGI (2021)-C scores with ordered iron deficiency stages. (**a**) Crude model; (**b**) model adjusted for age, sex, and BMI Z-score; (**c**) model further adjusted for boarding status, sleep duration, and physical activity. * *p* < 0.05, *** *p* < 0.001.

The associations of dietary patterns and CDGI (2021)-C with ordered stages of ID are shown in Figure 3. In the fully adjusted model, higher adherence to CDGI (2021)-C was inversely associated with the progression of ID stages (OR = 0.852, 95% CI: 0.751–0.966, *p*-trend = 0.012). Similar inverse associations were observed for the fruit–vegetable pattern (OR = 0.866, 95% CI: 0.748–0.993, *p*-trend = 0.047) and the meat–offal pattern (OR = 0.733, 95% CI: 0.611–0.868, *p*-trend < 0.001). In contrast, the snack–fast food pattern was associated with significantly higher odds of advancing ID stages (OR = 1.233, 95% CI: 1.094–1.381, *p*-trend < 0.001). No significant association was found for the cereal–tuber–legume pattern (OR = 1.019, 95% CI: 0.892–1.159, *p*-trend = 0.780).

### 3.4. Associations of Dietary Patterns and Dietary Index with Different Iron Deficiency Stages

#### 3.4.1. Associations of Dietary Patterns and Dietary Index with Early Stage Iron Deficiency

Table 3 shows the associations of dietary patterns and dietary index with early-stage iron deficiency (IDS). In Model 3 (fully adjusted), participants in the highest CDGI(2021)-C quartile (Q4) had a lower prevalence of IDS than Q1 (OR = 0.505, 95% CI: 0.315–0.808), with an inverse linear trend (*p*-trend = 0.027). The snack–fast food pattern was associated with a higher prevalence of IDS (Q4 vs. Q1: OR = 1.894, 95% CI: 1.249–2.872, *p*-trend = 0.040). Inverse associations were also observed for the fruit–vegetable pattern (Q4 vs. Q1: OR = 0.454, 95% CI: 0.282–0.732, *p*-trend = 0.049) and the meat–offal pattern (Q4 vs. Q1: OR = 0.538, 95% CI: 0.350–0.829, *p*-trend = 0.005). For the cereal–tuber–legume pattern, Q4 was associated with a higher prevalence of IDS (OR = 1.546, 95% CI: 1.009–2.369), but no significant linear trend was observed (*p*-trend > 0.05).

**Figure 4:**
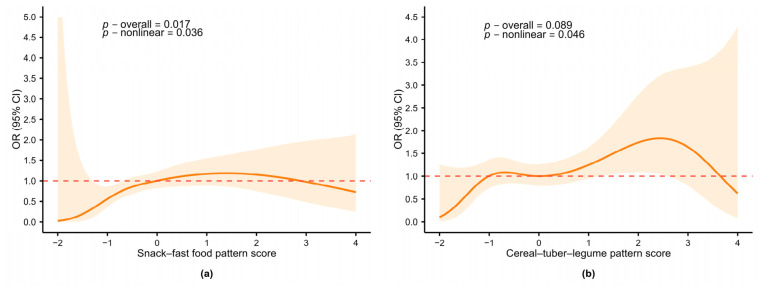
Restricted cubic spline analysis of dietary patterns in relation to IDS risk. (**a**) Snack–fast food pattern; (**b**) Cereal–tuber–legume pattern. Models were adjusted for age, sex, BMI Z-score, boarding status, father’s education level, and sleep duration. Solid line: OR; shaded area: 95% CI. Red dashed line at OR = 1: reference. OR: odds ratio; 95% CI: 95% confidence interval.

As shown in Table S5 and Figure 4, RCS analyses were used to examine the dose–response relationship. After full adjustment, the snack–fast food pattern exhibited a significant nonlinear association with IDS (*p*-overall = 0.017, *p*-nonlinear = 0.036). The risk curve rose initially and plateaued at moderate-to-high scores, peaking at approximately 1.5 (Figure 4a). The cereal–tuber–legume pattern showed a marginally significant overall association (*p*-overall = 0.089) but a significant nonlinear relationship (*p*-nonlinear = 0.046), with IDS risk increasing gradually and peaking within a score range of approximately 1–2.5 (Figure 4b). Consistent with Table 3, the fruit–vegetable pattern (*p*-overall = 0.013, *p*-linear = 0.005), meat–offal pattern (*p*-overall < 0.001, *p*-linear < 0.001), and CDGI(2021)-C score (*p*-overall < 0.001, *p*-linear < 0.001) were linearly associated with IDS, with no significant nonlinearity (*p*-nonlinear > 0.05).

#### 3.4.2. Associations of Dietary Patterns and Dietary Index with Middle-to-Late-Stage Iron Deficiency

As shown in Table 4, logistic regression analyses were performed to examine the associations of dietary patterns and dietary index with middle-to-late-stage iron deficiency (IDE and IDA). In the fully adjusted Model 3, only a per SD increase in the standardized snack–fast food dietary pattern score was significantly associated with higher risk (*p*-trend = 0.003).

**Figure 5:**
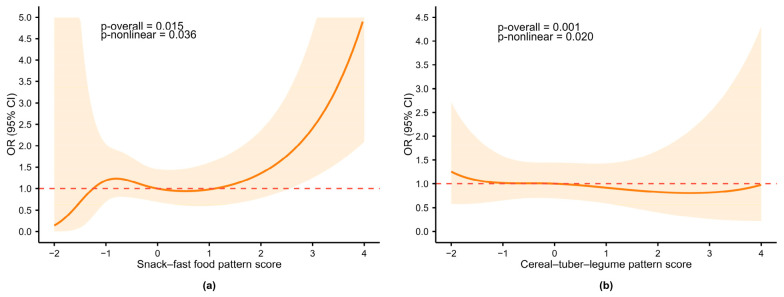
Restricted cubic spline analysis of dietary patterns in relation to middle-to-late-stage iron deficiency (IDE and IDA). (**a**) Snack–fast food pattern; (**b**) Cereal–tuber–legume pattern. Models were adjusted for age, sex, BMI Z-score, boarding status, moderate-to-high-intensity physical activity, and sleep duration. Solid line: OR; shaded area: 95% CI. Red dashed line at OR = 1: reference. OR: odds ratio; 95% CI: 95% confidence interval.

RCS regression analyses were used to explore dose–response relationships (Figure 5, Table S6). After adjustment, the snack–fast food pattern showed a significant overall association (*p*-overall = 0.015), with both linear (*p*-linear = 0.017) and nonlinear components (*p*-nonlinear = 0.036). As shown in Figure 5a, OR was stable near 1 for low-to-moderate scores (−1 to 1) and increased steeply above 1. The cereal–tuber–legume pattern showed a significant overall association (*p*-overall = 0.001), with linear (*p*-linear < 0.001) and nonlinear effects (*p*-nonlinear = 0.020). ORs remained close to 1, with a modest risk decrease at lower scores and a slight increase at higher scores (Figure 5b). For the meat–offal pattern, RCS showed a significant overall association (*p*-overall = 0.036) with a linear trend (*p*-linear = 0.023) and no nonlinearity (*p*-nonlinear = 0.280), although the per SD analysis was not significant (OR = 0.828, 95% CI: 0.617–1.111, *p*-trend = 0.208). No significant associations were observed for the fruit–vegetable pattern (*p*-overall = 0.365) or CDGI(2021)-C (*p*-overall = 0.362).

## 4. Discussion

Iron deficiency progresses through three stages, yet early subclinical phases remain poorly addressed. In our study, ID was dominated by subclinical IDS (68.22%), followed by IDE (17.45%) and IDA (14.33%), consistent with the natural progression of ID. Notably, most existing studies have focused on the overall prevalence of ID or IDA, with limited research on earlier stages. Compared with earlier studies in Beijing [36], the prevalence of IDS and IDE was lower, which may reflect regional and temporal differences in dietary intake and nutritional status. The rates of IDS and IDE were also lower than those in northwest China [37], possibly due to regional economic and dietary differences. Our IDA prevalence (1.85%) was lower than that among school-aged children in Europe (2.6%) [38] and far below the Chinese pooled prevalence (11.7%) [11], but higher than that in the U.S. (1.19%) [39], Turkey (1.62%) [40], and New Zealand (no IDA cases reported) [41]. These variations may be explained by differences in age, ethnicity, economy, nutrition, and diagnostic criteria. Although IDA prevalence was low, the high proportion of subclinical ID indicates that ID in children and adolescents still warrants attention.

Older age, female sex, and shorter sleep duration were associated with a more severe ID status. The increased iron demand during adolescent growth, menstrual iron loss in girls, and impaired iron metabolism related to poor sleep might have contributed to these associations [11,42,43,44,45]. Importantly, the proportion of females gradually increased from IDS to IDE and IDA, suggesting that persistent iron loss and insufficient dietary intake may promote the progression of ID [11,44,45]. ID status varied significantly by nutritional status, with normal-weight children and adolescents having the highest prevalence of advancing ID stages. Most studies report a higher risk of ID in obese populations, attributed to chronic inflammation that elevates hepcidin and inhibits iron absorption [46]. However, evidence among children and adolescents remains inconsistent due to variations in diagnostic criteria, population characteristics, and sample sizes [47]. Overweight/obese school-aged children mainly exhibited excessive intake of high-calorie foods, alongside relatively adequate consumption of animal-sourced foods and dietary iron. Additionally, overweight/obesity in this region is predominantly metabolically healthy, accompanied by mild inflammation [48]. These factors may contribute to our findings. Boarding students had a higher risk of moderate-to-severe ID, possibly reflecting limited dietary variety and irregular meals. Insufficient physical activity was linked to more severe ID. Appropriate physical activity may enhance iron utilization by reducing inflammatory cytokines and hepatic hepcidin expression [49]. Dietary factors play a key role in iron homeostasis. This study further explored their associations with ID across different stages by using both dietary index and dietary patterns.

In this study, the dietary index was assessed using the CDGI(2021)-C. The CDGI(2021)-C is a validated index based on the Chinese Dietary Guidelines (2016) and has been shown to reliably evaluate dietary quality among Chinese children and adolescents aged 7–17 years [21]. It has also been applied to explore diet-related health outcomes, such as bone mineral density, in school-aged children [22]. In the present study, higher CDGI(2021)-C adherence was inversely associated with ID severity, suggesting that diets more consistent with national dietary guidelines were related to better iron status. These findings are consistent with previous studies reporting that higher diet quality indices, including the HEI and KIDMED, were associated with favorable iron status and lower ID risk in children and adults [19,20]. Although some studies found no significant association between overall diet quality indices and iron status, significant relations were observed for specific food groups [50]. Notably, evidence linking dietary indices to ID stages remained scarce. To our knowledge, the present study was the first to explore the associations between CDGI(2021)-C and different stages of ID in school-aged children.

CDGI(2021)-C was introduced as a complementary dietary indicator to strengthen the evidence base of the observed associations. To validate its convergent validity with the four empirically derived dietary patterns, we examined their associations. The CDGI(2021)-C score was positively associated with all four dietary patterns, with marked variation in the strength of these associations. The fruit–vegetable and cereal–tuber–legume patterns showed the strongest correlations, consistent with dietary guidelines that emphasize plant-based foods as the basis of high-quality diets. The weak but significant positive correlation for the snack–fast food pattern may be partly explained by nutrient-dense components such as dairy and nuts within this pattern, as well as potential residual confounding by socioeconomic status. Higher-income individuals often maintain better overall diet quality but may also consume more processed and convenient foods. The very weak correlation for the meat–offal pattern mainly reflected the index’s bidirectional scoring system, which favors moderate intake and penalizes both insufficient and excessive consumption. Collectively, these results indicated that plant-dominant patterns were most consistent with high dietary quality, while the weaker associations for other patterns highlighted the complexity of dietary structures and the importance of considering pattern composition in dietary quality assessment.

Our ordered logistic regression analysis identified consistent associations between dietary indices and ID severity. Higher CDGI(2021)-C, fruit–vegetable, and meat–offal pattern scores were linked to lower odds of advancing ID stages, while the snack–fast food pattern showed adverse effects. These findings extend our previous binary results [25], showing that dietary effects manifest at subclinical stages and persist to anemia. The cereal–tuber–legume pattern was not significantly linked to stage severity. Its earlier positive association with overall ID may be restricted to early stages or confounded by other dietary factors. Dose–response trends for CDGI(2021)-C and the fruit–vegetable pattern indicated that higher diet quality and plant diversity offer graded protection against worsening ID. Although mechanisms like vitamin C-enhanced iron absorption and heme iron bioavailability are well known [51], our study confirms that these protective associations extend across all ID stages.

Consistent with the overall dose–response trends from ordered logistic regression, dietary effects were more pronounced in the IDS stage. Both quartile logistic regression and RCS analyses showed that CDGI(2021)-C, fruit–vegetable pattern, and the meat–offal pattern were linearly and inversely associated with IDS risk. In contrast, the snack–fast food pattern exhibited a significant nonlinear association: risk initially increased, plateaued, and peaked at about 1.5, consistent with the quartile results. The cereal–tuber–legume pattern also showed a significant nonlinear relationship, with risk stable at low scores, peaking at moderate scores, and declining at higher scores. This may involve the displacement of iron-rich foods and the inhibition of non-heme iron absorption by antinutritional factors like phytic acid and tannins [52,53]. The decreased risk at very high scores might be due to the limited sample size, warranting confirmation in larger studies.

In middle-to-late-stage ID, dietary effects were markedly attenuated, further confirming that the overall protective trends from ordered logistic regression were driven mainly by the early stage (IDS). The snack–fast food pattern, however, showed a persistent adverse effect with a clear threshold: risk remained stable at low-to-moderate scores and rose steeply above 1. This pattern is rich in snacks, fast foods, and sugar-sweetened beverages and low in iron but high in energy, fat, added sugar, and sodium. Long-term consumption may disturb iron metabolism, reduce ferritin, and promote ID progression [54,55,56]. Sugar-sweetened beverages also contribute to inadequate iron intake in children [57]. The cereal–tuber–legume pattern still showed a significant nonlinear association at this stage, but the curve flattened, with ORs close to 1. This weakening may be related to sample limitations, physiological changes, or masking of anti-nutritional effects by advanced metabolic disturbances. For the meat–offal pattern, a linear trend was observed in RCS but not in the per-SD analysis, indicating weak overall effects. Once iron depletion has advanced, dietary intervention alone may be insufficient to reverse the deficiency, and inflammation or chronic diseases may further mask dietary effects [58].

To our knowledge, this is the first study to combine dietary pattern analysis with CDGI(2021)-C to explore associations between dietary factors and ID stages, and to confirm consistent results between the two indicators. However, several limitations should be noted. First, the cross-sectional design precludes causal inference between dietary factors and ID progression across different stages. Second, the assessment of ID-related biomarkers was relatively limited. Third, the FFQ is prone to recall bias, and one-month dietary data may not fully reflect long-term dietary habits. Moreover, since we used a food group–based FFQ without item-specific portion sizes, we could not estimate quantitative dietary iron intake. Fourth, the study was restricted to rural children and adolescents in Guangzhou. The identified dietary patterns present regional specificity, so caution is needed when extrapolating these findings. Future long-term prospective cohort studies with multi-indicator measurements and intervention trials are needed to verify our results.

## 5. Conclusions

Subclinical ID accounted for the majority of ID cases among children and adolescents. This study examined the associations of dietary patterns and dietary indices with ID severity across different stages. Both dietary assessment approaches showed coherent associations across ID stages. Higher adherence to the CDGI(2021)-C, fruit–vegetable pattern, and meat–offal pattern was associated with lower odds of advancing ID stages, while the snack–fast food pattern showed the opposite trend. These protective or harmful effects were evident at the IDS stage and attenuated in IDE/IDA. Restricting snack and fast-food intake still conferred long-term benefits due to its persistent risk across stages. These findings highlight the importance of stage-specific dietary interventions, with early-stage ID as a critical window for targeted dietary modification.

## Figures and Tables

**Table 1:** The general characteristics of study participants by iron deficiency status.

Variables	Non-ID (*n* = 2172)	IDS (*n* = 219)	IDE (*n* = 56)	IDA (*n* = 46)	*p*
Age, median (IQR)	13.16 (11.12, 14.35)	13.32 (12.23, 14.34)	13.88 (13.24, 14.49)	14.38 (13.56, 15.66)	**<0.001**
sex, *n* (%)					**<0.001**
Male	1273 (94.51%)	59 (4.38%)	14 (1.04%)	1 (0.07%)	
Female	899 (78.45%)	160 (13.96%)	42 (3.66%)	45 (3.93%)	
BMI category (WHO standards), *n* (%)					**<0.001**
Malnutrition	126 (91.30%)	7 (5.07%)	3 (2.17%)	2 (1.45%)	
Normal	1618 (85.29%)	194 (10.23%)	45 (2.37%)	40 (2.11%)	
Overweight or obesity	428 (93.45%)	18 (3.93%)	8 (1.75%)	4 (0.87%)	
Boarding, *n* (%)					**<0.001**
Yes	1056 (86.49%)	91 (7.45%)	40 (3.28%)	34 (2.78%)	
No	1116 (87.74%)	128 (10.06%)	16 (1.26%)	12 (0.94%)	
Education of father, *n* (%)					0.167
Primary school or below	75 (87.21%)	8 (9.30%)	3(3.49%)	0 (0.00%)	
Middle school	867 (85.25%)	110 (10.82%)	20 (1.97%)	20 (1.97%)	
High school	629 (87.48%)	55 (7.65%)	20 (2.78%)	15 (2.09%)	
College degree or above	540 (89.11%)	44 (7.26%)	11 (1.82%)	11 (1.82%)	
Unknown	61 (93.85%)	2 (3.08%)	2 (3.08%)	0 (0.00%)	
Education of mother, *n* (%)					0.116
Primary school or below	113 (84.96%)	16 (12.03%)	4 (3.01%)	0 (0.00%)	
Middle school	929 (85.62%)	109 (10.05%)	21 (1.94%)	26 (2.40%)	
High school	526 (87.09%)	51 (8.44%)	18 (2.98%)	9 (1.49%)	
College degree or above	550 (89.87%)	40 (6.54%)	11 (1.80%)	11 (1.80%)	
Unknown	54 (91.53%)	3 (5.08%)	2 (3.39%)	0 (0.00%)	
Sleep duration, median (IQR)	9.09 (8.17, 10.00)	8.75 (8.00, 9.67)	8.67 (8.00, 9.37)	8.25 (7.71, 9.00)	**<0.001**
Moderate-to-high-intensity physical activity, *n* (%)					**0.027**
<3 times/week	989 (87.06%)	88 (7.75%)	34 (2.99%)	25 (2.20%)	
≥3 times/week	1183 (87.18%)	131 (9.65%)	22 (1.62%)	21 (1.55%)	
Smoking attempt, *n* (%)					0.610
Yes	133 (91.10%)	10 (6.85%)	2 (1.37%)	1 (0.68%)	
No	2039 (86.88%)	209 (8.90%)	54 (2.30%)	45 (1.92%)	
Alcohol consumption, *n* (%)					0.501
Yes	307 (85.04%)	39 (10.80%)	9 (2.49%)	6 (1.66%)	
No	1865 (87.48%)	180 (8.44%)	47 (2.20%)	40 (1.88%)	
CDGI(2021)-C, median (IQR)	44.22 (36.23, 51.87)	40.76 (33.38, 47.67)	41.98 (36.27, 48.67)	38.90 (34.19, 48.35)	**<0.001**

Note: Non-ID: non-iron deficiency. IDS: iron deficiency store. IDE: iron deficient erythropoiesis. IDA: iron deficiency anemia. CDGI(2021)-C: Chinese Dietary Guidelines Index for Children and Adolescents Aged 7–17 (2021). Data were presented as median (P_25_, P_75_) or *n* (%). The Kruskal–Wallis test was used for non-normally distributed continuous variables, and the chi-Squared test or Fisher’s exact test for categorical variables. Bold *p*-values indicate “<0.05”.

**Table 2:** Spearman correlation coefficients between dietary pattern scores and CDGI(2021)-C Scores.

Dietary Pattern	Spearman’s *r_s_* (95% CI)	*p*
Snack–fast food pattern	0.168 (0.126, 0.209)	**<0.001**
Fruit–vegetable pattern	0.552 (0.524, 0.581)	**<0.001**
Cereal–tuber–legume pattern	0.386 (0.350, 0.419)	**<0.001**
Meat–offal pattern	0.068 (0.028, 0.110)	**<0.001**

Note: Spearman correlation analysis was conducted to examine the associations between dietary pattern scores and CDGI(2021)-C scores. *r_s_*: Spearman correlation coefficient; 95% CI: 95% confidence interval. Bold *p*-values indicate “<0.05”.

**Table 3:** Association of dietary patterns and dietary index with early stage iron deficiency (IDS).

Dietary Factors	Model 1		Model 2		Model 3	
	OR (95%CI)	*p*	OR (95%CI)	*p*	OR (95%CI)	*p*
CDGI(2021)-C						
Q1	1		1		1	
Q2	0.868 (0.602–1.253)	0.450	0.941 (0.647–1.369)	0.750	0.981 (0.671–1.436)	0.923
Q3	0.920 (0.640–1.321)	0.651	1.154 (0.792–1.681)	0.457	1.232 (0.838–1.813)	0.289
Q4	0.383 (0.243–0.604)	**<0.001**	0.466 (0.291–0.746)	**0.001**	0.505 (0.315–0.808)	**0.004**
*p*-trend	0.761 (0.664–0.872)	**<0.001**	0.823 (0.713–0.949)	**0.008**	0.850 (0.736–0.982)	**0.027**
Snack–fast food pattern						
Q1	1		1		1	
Q2	1.546 (1.024–2.335)	**0.038**	1.515 (0.997–2.304)	0.052	1.530 (1.001–2.339)	**0.049**
Q3	1.268 (0.827–1.943)	0.276	1.302 (0.847–2.003)	0.229	1.312 (0.850–2.024)	0.221
Q4	1.682 (1.120–2.526)	**0.012**	1.882 (1.246–2.843)	**0.003**	1.894 (1.249–2.872)	**0.003**
*p*-trend	1.069 (0.962–1.189)	0.213	1.121 (1.002–1.255)	**0.045**	1.123 (1.005–1.256)	**0.040**
Frui–vegetable pattern						
Q1	1		1		1	
Q2	0.950 (0.656–1.375)	0.785	0.856 (0.588–1.247)	0.418	0.891 (0.608–1.305)	0.553
Q3	1.019 (0.708–1.468)	0.918	0.873 (0.599–1.273)	0.481	0.909 (0.619–1.334)	0.626
Q4	0.427 (0.271–0.672)	**<0.001**	0.399 (0.250–0.635)	**<0.001**	0.454 (0.282–0.732)	**0.001**
*p*-trend	0.801 (0.669–0.959)	**0.016**	0.774 (0.631–0.949)	**0.014**	0.813 (0.662–0.999)	**0.049**
Cereal–tuber–legume pattern						
Q1	1		1		1	
Q2	1.162 (0.793–1.705)	0.441	1.330 (0.898–1.968)	0.154	1.323 (0.892–1.963)	0.164
Q3	0.919 (0.615–1.374)	0.682	1.189 (0.788–1.793)	0.410	1.190 (0.786–1.802)	0.411
Q4	0.980 (0.659–1.456)	0.919	1.562 (1.027–2.374)	**0.037**	1.546 (1.009–2.369)	**0.045**
*p*-trend	0.988 (0.866–1.127)	0.855	1.147 (1.009–1.305)	**0.037**	1.143 (0.999–1.308)	0.053
Meat–offal pattern						
Q1	1		1		1	
Q2	0.712 (0.495–1.025)	0.068	0.740 (0.509–1.076)	0.115	0.720 (0.493–1.053)	0.090
Q3	0.629 (0.432–0.916)	**0.016**	0.682 (0.464–1.002)	0.051	0.662 (0.448–0.977)	**0.038**
Q4	0.455 (0.302–0.686)	**<0.001**	0.550 (0.358–0.845)	**0.006**	0.538 (0.350–0.829)	**0.005**
*p*-trend	0.689 (0.569–0.835)	**<0.001**	0.734 (0.595–0.906)	**0.004**	0.729 (0.585–0.909)	**0.005**

Note: Multivariable logistic regression was used to estimate odds ratios (ORs) and 95% confidence intervals (CIs) for early stage iron deficiency (IDS) by quartiles of dietary factor scores, with Q1 as reference. *p*-trend values represent per 1-SD increase in standardized scores. Model 1: unadjusted; Model 2: adjusted for age, sex, BMI Z-score; Model 3: further adjusted for boarding status, father education level, and sleep duration. The bold *p*-value means “<0.05”.

**Table 4:** Associations of dietary patterns and dietary index with middle-to-late-stage iron deficiency (IDE and IDA).

Dietary Factors	Model 1		Model 2		Model 3	
	OR (95%CI)	*p*	OR (95%CI)	*p*	OR (95%CI)	*p*
CDGI(2021)-C						
Q1	1		1		1	
Q2	1.180 (0.710–1.962)	0.523	1.441 (0.853–2.433)	0.172	1.437 (0.847–2.438)	0.179
Q3	0.751 (0.427–1.322)	0.321	1.062 (0.591–1.908)	0.840	1.071 (0.595–1.930)	0.819
Q4	0.574 (0.312–1.055)	0.074	0.949 (0.502–1.792)	0.871	0.962 (0.507–1.827)	0.906
*p*-trend	0.800 (0.667–0.958)	**0.016**	0.951 (0.780–1.161)	0.623	0.957 (0.782–1.170)	0.665
Snack–fast food pattern						
Q1	1		1		1	
Q2	1.045 (0.597–1.831)	0.877	0.929 (0.525–1.643)	0.799	0.896 (0.506–1.587)	0.707
Q3	0.918 (0.516–1.636)	0.773	0.872 (0.479–1.585)	0.652	0.838 (0.459–1.531)	0.566
Q4	1.126 (0.649–1.953)	0.674	1.206 (0.684–2.127)	0.518	1.133 (0.641–2.003)	0.667
*p*-trend	1.223 (1.033–1.449)	**0.020**	1.365 (1.126–1.655)	**0.002**	1.337 (1.103–1.621)	**0.003**
Fruit–vegetable pattern						
Q1	1		1		1	
Q2	1.158 (0.629–2.130)	0.638	1.014 (0.543–1.894)	0.965	1.040 (0.555–1.947)	0.903
Q3	1.798 (1.026–3.150)	**0.040**	1.507 (0.841–2.701)	0.168	1.535 (0.853–2.761)	0.153
Q4	1.210 (0.661–2.214)	0.536	1.140 (0.612–2.124)	0.679	1.122 (0.600–2.098)	0.718
*p*-trend	0.996 (0.841–1.180)	0.964	0.992 (0.813–1.210)	0.936	0.983 (0.804–1.202)	0.864
Cereal–tuber–legume pattern						
Q1	1		1		1	
Q2	0.716 (0.431–1.192)	0.199	0.763 (0.452–1.288)	0.311	0.778 (0.460–1.317)	0.350
Q3	0.471 (0.265–0.837)	**0.010**	0.620 (0.344–1.118)	0.112	0.639 (0.353–1.158)	0.140
Q4	0.525 (0.301–0.916)	**0.023**	0.903 (0.499–1.633)	0.735	0.974 (0.537–1.767)	0.932
*p*-trend	0.679 (0.520–0.886)	**0.004**	0.811 (0.572–1.150)	0.240	0.834 (0.595–1.170)	0.294
Meat–offal pattern						
Q1	1		1		1	
Q2	0.740 (0.444–1.234)	0.249	0.817 (0.480–1.393)	0.458	0.837 (0.489–1.435)	0.519
Q3	0.570 (0.329–0.988)	**0.045**	0.627 (0.351–1.118)	0.113	0.632 (0.353–1.133)	0.123
Q4	0.486 (0.273–0.865)	**0.014**	0.621 (0.327–1.178)	0.145	0.633 (0.332–1.206)	0.164
*p*-trend	0.756 (0.589–0.971)	**0.028**	0.823 (0.609–1.114)	0.207	0.828 (0.617–1.111)	0.208

Note: Multivariable logistic regression was used to estimate odds ratios (ORs) and 95% CIs for middle-to-late-stage iron deficiency (IDE and IDA) by quartiles of dietary factor scores, with Q1 as reference. *p*-trend values represent per 1-SD increase in standardized adherence scores. Model 1: unadjusted; Model 2: adjusted for age, sex, BMI Z-score; Model 3: further adjusted for boarding status, moderate-to-high-intensity physical activity and sleep duration. The bold *p*-value means “<0.05”.

## Data Availability

The data presented in this study are available on request from the corresponding author due to privacy.

## Supplementary Materials

The following supporting information can be downloaded at: https://www.mdpi.com/article/10.3390/nu18101620/s1, Table S1: Evaluation indicators and scoring methods of the Chinese Dietary Guidelines Index for children and adolescents aged 7–17 (2021); Table S2. Food groups used in the factor analysis; Table S3. Characteristics of dietary pattern score quartiles (Q) in study participants; Table S4. Characteristics of CDGI(2021)-C score quartiles (Q) in study participants; Table S5. Summary of Restricted Cubic Spline Analyses of Associations Between Dietary Patterns, Dietary Index, and early stage of iron deficiency (IDS); Table S6. Summary of Restricted Cubic Spline Analyses of Associations Between Dietary Patterns, Dietary Index, and middle-to-late-stage iron deficiency (IDE and IDA).

## Abbreviations

The following abbreviations are used in this manuscript:

ID	Iron deficiency
IDS	Iron deficiency store
IDE	Iron deficiency erythropoiesis
IDA	Iron deficiency anemia
CDGI(2021)-C	The Chinese Dietary Guidelines Index for Children
FFQ	Food frequency questionnaire
BMI	Body mass index
SF	Serum ferritin
CRP	C-reactive protein
SI	Serum iron
TIBC	Total iron-binding capacity
Hb	Hemoglobin
TS	Transferrin saturation
SD	Standard deviation
IQR	Interquartile range
PR	Prevalence ratio
OR	Odds ratio
CI	Confidence interval
RCS	Restricted cubic spline
HEI	Healthy Eating Index
DQI	Diet Quality Index
KIDMED	Mediterranean Diet Quality Index for children and adolescents
WHO	World Health Organization
GZCDC	Guangzhou Center for Disease Control and Prevention
