# Supplementary material for: Associations of Dietary Patterns and Dietary Index with Iron Deficiency Across Different Stages Among Children Aged 9–17 Years in Guangzhou, China: A Cross-Sectional Study
Source: Nutrients. 2026 May 20;18(10):1620. doi: 10.3390/nu18101620 (PMC13210202; doi:10.3390/nu18101620)
Supplement: Supplementary file 1 [file nutrients-18-01620-s001.zip › nutrients-4229281-supplementary.pdf]

Table S1. Evaluation indicators and scoring methods of the Chinese Dietary Guidelines Index for children and adolescents aged 7-17 (2021).

Age	Dietary index	Recommended amount	Minimum standard zero score	Maximum criterion	Maximum assignment
7–10 years old	Carbohydrate supply ratio	50%–65%	0% or 100%	50%–65%	5
	Tubers	30–70 g/d	0 g/d	≥50 g/d	5
	Vegetables	300 g/d	0 g/d	≥300 g/d	5
	Fruits	150–200 g/d	0 g/d	≥175 g/d	10
	Dairy products	300 g/d	0 g/d	≥300 g/d	10
	Beans	15 g/d	0 g/d	≥15 g/d	5
	Nuts			≥0 g/d	5
	Meats	40 g/d	0 g/d or ≥80g/d	40 g/d	10
	Eggs	25–40 g/d	0 g/d or ≥65g/d	25–40 g/d	10
	Aquatic products	40g/d	0 g/d	≥40 g/d	10
11–13 years old	Carbohydrate supply ratio	50%–65%	0 % or 100%	50%–65%	5
	Tubers	30–70 g/d	0 g/d	≥50 g/d	5
	Vegetables	400–450 g/d	0 g/d	≥425 g/d	5
	Fruits	200–300 g/d	0 g/d	≥250 g/d	10
	Dairy products	300 g/d	0 g/d	≥300 g/d	10
	Beans	15 g/d	0 g/d	≥15 g/d	5
	Nuts	7–10 g/d	0 g/d	≥8.5 g/d	5
	Meats	50 g/d	0 g/d or ≥100 g/d	50 g/d	10
	Eggs	40–50 g/d	0 g/d or ≥90 g/d	40–50 g/d	10
	Aquatic products	50 g/d	0 g/d	≥50 g/d	10
14–17 years old	Carbohydrate supply ratio	50%–65%	0% 或 100%	50%–65%	5
	Tubers	50–100 g / d	0 g / d	≥75 g / d	5
	Vegetables	450–500 g / d	0 g / d	≥475 g / d	5

Age	Dietary index	Recommended amount	Minimum standard zero score	Maximum criterion	Maximum assignment
	Fruits	300–350 g / d	0 g / d	≥325 g / d	10
	Dairy products	300 g / d	0 g / d	≥300 g / d	10
	Beans	15 –25 g / d	0 g / d	≥20 g / d	5
	Nuts	7–10 g / d	0 g / d	≥8. 5 g / d	5
	Meats	50–75 g / d	0 g / d or ≥125 g / d	50–75 g / d	10
	Eggs	50 g / d	0 g / d or ≥100 g / d	50 g / d	10
	Aquatic products	50–75 g / d	0 g / d	≥62. 5 g / d	10

Table S2. Food groups used in the factor analysis.

Number	Food category	Examples of Food Items
1	Cereals and Tubers	Rice and rice products, wheat and wheat products, corn, potato, sweet potato
2	Soybeans and their products	Soybean, soy milk, tofu, dried bean curd
3	Fresh vegetables	Lettuce, tomato, broccoli, bok choy, cucumber
4	Mushrooms and algae	Mushroom, laver, kelp
5	Fresh fruits	Apple, banana, pear, orange
6	Dairy and dairy products	milk, goat's milk, yogurt, cheese, milk tablet
7	Red meat	pork, beef, lamb, bacon, ham
8	Poultry	Chicken, duck, goose
9	Animal offal	Heart, liver, kidney, large intestine
10	Aquatic products	Fish, shrimp, crab, clam
11	Eggs and their products	Egg, duck egg, quail egg
12	Nuts	Peanut, almond, walnut, hazelnut
13	Candies	Sugar, jam, jelly, candy, chocolate
14	Snack food	Spicy strip, potato chip, and fried puffed snack
15	Fast food	Instant noodle, self-heating rice, hamburger
16	Beverages	Milk tea, dairy-containing beverage, tea beverage, sports beverage

Table S3. Characteristics of dietary pattern score quartiles (Q) in study participants.

**Variables**	**Snack−fast food pattern**		***p***	**Fruit−vegetable pattern**		***p***	**Cereal−tuber-legume pattern**		***p***	**Meat−offal pattern**		***p***
	**Q1**	**Q4**		**Q1**	**Q4**		**Q1**	**Q4**		**Q1**	**Q4**	
Age, median (IQR)	12.25 (10.82,14.26)	13.41 (11.58,14.46)	**<0.001**	13.44 (11.62,14.47)	13.11 (10.82,14.34)	**<0.001**	12.76 (10.93,14.18)	13.37 (11.38,14.46)	**<0.001**	12.98 (10.93,14.16)	13.67 (11.59,15.32)	**<0.001**
sex, n (%)			**<0.001**			**0.013**			**<0.001**			**<0.001**
Male	303(43.85%)	388 (56.15%)		389 (53.07%)	344 (46.93%)		228 (33.83%)	446 (66.17%)		274 (39.60%)	418 (60.40%)	
Female	321(57.63%)	236 (42.37%)		235 (45.72%)	279 (54.28%)		395 (69.06%)	177 (30.94%)		350 (63.06%)	205 (36.94%)	
BMI category (WHO standards)			**0.045**			0.480			**0.007**			0.104
Malnutrition	36 (51.43%)	34 (48.57%)		34 (46.58%)	39 (53.42%)		25 (35.71%)	45 (64.29%)		36 (61.02%)	23 (38.98%)	
Normal	459 (48.16%)	494(51.84%)		483 (51.00%)	464 (49.00%)		494 (52.28%)	451 (47.72%)		473 (50.43%)	465 (49.57%)	
Overweight or obesity	129 (57.33%)	96(42.67%)		107 (47.14%)	120 (52.86%)		104 (45.02%)	127 (54.98%)		115 (46.00%)	135 (54.00%)	
Boarding, n (%)			**<0.001**			0.380			0.571			**0.010**
Yes	256 (44.21%)	323 (55.79%)		297 (48.69%)	313 (51.31%)		303 (50.92%)	292 (49.08%)		287 (46.37%)	332 (53.63%)	
No	368 (55.01%)	301 (44.99%)		327 (51.33%)	310 (48.67%)		320 (49.16%)	331 (50.84%)		337 (53.66%)	291 (46.34%)	
Education of father, n (%)			**0.003**			**0.019**			0. 253			**0.013**
Primary school or below	12 (32.43%)	25 (67.57%)		22 (52.38%)	20 (47.62%)		19 (50.00%)	19 (50.00%)		17 (44.74%)	21 (55.26%)	
Middle school	233 (45.96%)	274 (54.04%)		273 (55.38%)	220 (44.62%)		256 (51.51%)	241 (48.49%)		271 (54.75%)	224 (45.25%)	
High school	185 (52.11%)	170 (47.89%)		167 (46.26%)	194 (53.74%)		176 (48.09%)	190 (51.91%)		184 (50.00%)	184 (50.00%)	
College degree or above	181	136		140	172		151	163		135	182	

Variables	Snack−fast food pattern		*p*	Fruit−vegetable pattern		*p*	Cereal−tuber-legume pattern		*p*	Meat−offal pattern		*p*
	Q1	Q4		Q1	Q4		Q1	Q4		Q1	Q4	
	(57.10%)	(42.90%)		(44.87%)	(55.13%)		(48.09%)	(51.91%)		(42.59%)	(57.41%)	
Unknown	13 (40.62%)	19 (59.38%)		22 (56.41%)	17 (43.59%)		21 (67.74%)	10 (32.26%)		17 (58.62%)	12 (41.38%)	
Education of mother, n (%)			**0.029**			**0.003**			0.410			**0.009**
Primary school or below	29 (45.31%)	35 (54.69%)		39 (61.90%)	24 (38.10%)		33 (52.38%)	30 (47.62%)		31 (50.00%)	31 (50.00%)	
Middle school	250 (46.64%)	286 (53.36%)		283 (54.95%)	232 (45.05%)		266 (49.53%)	271 (50.47%)		297 (54.80%)	245 (45.20%)	
High school	158 (51.47%)	149 (48.53%)		137 (43.63%)	177 (56.37%)		159 (51.79%)	148 (48.21%)		142 (48.80%)	149 (51.20%)	
College degree or above	176 (56.59%)	135 (43.41%)		147 (45.79%)	174 (54.21%)		148 (47.28%)	165 (52.72%)		138 (42.46%)	187 (57.54%)	
Unknown	11 (36.67%)	19 (63.33%)		18 (52.94%)	16 (47.06%)		17 (65.38%)	9 (34.62%)		16 (59.26%)	11 (40.74%)	
Sleep duration, median (IQR)	9.33 (8.33,10.17)	9.00 (8.00,9.69)	**<0.001**	9.00 (8.00,9.87)	9.25 (8.33,10.17)	**<0.001**	9.17 (8.25,10.00)	9.00 (8.00,10.00)	0.486	9.33 (8.25,10.00)	8.83 (8.00,9.83)	**<0.001**
Moderate-to-high-intensity physical activity, n (%)			**0.003**			0.349			**<0.001**			0.199
<3 times/week	298 (54.88%)	245 (45.12%)		270 (48.47%)	287 (51.53%)		348 (60.00%)	232 (40.00%)		292 (52.05%)	269 (47.95%)	
≥3 times/week	326 (46.24%)	379 (53.76%)		354 (51.30%)	336 (48.70%)		275 (41.29%)	391 (58.71%)		332 (48.40%)	354 (51.60%)	
Attempt smoking, n (%)			**<0.001**			**0.001**			0.807			0.994
Yes	12 (17.14%)	58 (82.86%)		55 (67.90%)	26 (32.10%)		34 (47.89%)	37 (52.11%)		40 (50.00%)	40 (50.00%)	
No	612 (51.95%)	566 (48.05%)		569 (48.80%)	597 (51.20%)		589 (50.13%)	586 (49.87%)		584 (50.04%)	583 (49.96%)	
Alcohol consumption, n (%)			**<0.001**			**0.040**			0.468			0.051
Yes	49 (26.49%)	136 (73.51%)		107 (57.22%)	80 (42.78%)		85 (47.22%)	95 (52.78%)		86 (43.65%)	111 (56.35%)	
No	575	488		517	543		538	528		538	512	

Variables	Snack–fast food pattern		*p*	Fruit–vegetable pattern		*p*	Cereal–tuber-legume pattern		*p*	Meat–offal pattern		*p*
	**Q1**	**Q4**		**Q1**	**Q4**		**Q1**	**Q4**		**Q1**	**Q4**	
	(54.09%)	(45.91%)		(48.77%)	(51.23%)		(50.47%)	(49.53%)		(51.24%)	(48.76%)	
CDGI(2021)-C, n (%)			**<0.001**			**<0.001**			**<0.001**			**0.008**
Q1	180 (67.67%)	86 (32.33%)		285 (93.44%)	20 (6.56%)		266 (84.44%)	49 (15.56%)		197 (57.10%)	148 (42.90%)	
Q2	151 (55.31%)	122 (44.69%)		184 (69.70%)	80 (30.30%)		175 (61.19%)	111 (38.81%)		138 (50.18%)	137 (49.82%)	
Q3	134 (41.10%)	192 (58.90%)		123 (42.41%)	167 (57.59%)		100 (32.47%)	208 (67.53%)		130 (43.92%)	166 (56.08%)	
Q4	159 (41.51%)	224 (58.49%)		32 (8.25%)	356 (91.75%)		82 (24.33%)	255 (75.67%)		159 (48.04%)	172 (51.96%)	

Note: Q1: the lowest consumption of each pattern; Q4: the highest intake of the food pattern. Data was presented as median (P_{25}, P_{75}) or n (%). Mann–Whitney U test for non-normally distributed variables and Chi-Squared test for categorical variables. Bold *p*-values indicate " <0.05".

Table S4. Characteristics of CDGI(2021)-C score quartiles (Q) in study participants.

Variables	CDGI(2021)-C		*p*
	Q1	Q4	
Age, median (IQR)	13.43 (11.59,14.66)	12.59 (10.71,14.00)	**<0.001**
sex, n (%)			**<0.001**
Male	286 (43.66%)	369 (56.34%)	
Female	337 (56.93%)	255 (43.07%)	
BMI category (WHO standards)			0.204
Malnutrition	26 (39.39%)	40 (60.61%)	
Normal	487 (50.73%)	473 (49.27%)	
Overweight or obesity	110 (49.77%)	111 (50.23%)	
Boarding, n (%)			**0.014**
Yes	318 (53.72%)	274 (46.28%)	
No	305 (46.56%)	350 (53.44%)	
Education of father, n (%)			**0.003**
Primary school or below	25 (62.50%)	15 (37.50%)	
Middle school	285 (55.13%)	232 (44.87%)	
High school	158 (46.61%)	181 (53.39%)	
College degree or above	136 (43.04%)	180 (56.96%)	
Unknown	19 (54.29%)	16 (45.71%)	
Education of mother, n (%)			**<0.001**
Primary school or below	43 (67.19%)	21 (32.81%)	
Middle school	311 (55.73%)	247 (44.27%)	
High school	124 (44.29%)	156 (55.71%)	
College degree or above	129 (41.21%)	184 (58.79%)	
Unknown	16 (50.00%)	16 (50.00%)	
Sleep duration, median (IQR)	9.00(8.00,10.00)	9.50(8.50,10.27)	**<0.001**
Moderate-to-high-intensity physical activity, n (%)			**<0.001**
<3 times/week	331 (56.39%)	256 (43.61%)	
≥3 times/week	292 (44.24%)	368 (55.76%)	
Attempt smoking, n (%)			0.219
Yes	41 (57.75%)	30 (42.25%)	
No	582 (49.49%)	594 (50.51%)	
Alcohol consumption, n (%)			0.798
Yes	90 (51.14%)	86 (48.86%)	
No	533 (49.77%)	538 (50.23%)	

Note: Q1 and Q4 represent the lowest and highest quartiles of CDGI(2021)-C score, respectively. Data was presented as median (P_{25}, P_{75}) or n (%). Mann–Whitney U test for non-normally distributed variables and Chi-Squared test for categorical variables. Bold *p*-values indicate " <0.05".

Table S5. Summary of Restricted Cubic Spline Analyses of Associations Between Dietary Patterns, Dietary Index, and early stage of iron deficiency (IDS).

Variable	*p*-overall	*p*-linear	*p*-nonlinear
CDGI (2021)-C score	**<0.001**	**<0.001**	0.071
Snack–fast food pattern score	**0.017**	0.319	**0.036**
Cereal–tuber–legume pattern score	0.089	0.863	**0.046**
Meat–offal pattern score	**<0.001**	**<0.001**	0.155
Fruit–vegetable pattern score	**0.013**	**0.005**	0.291

Note: Restricted cubic spline (RCS) regression was used to evaluate the dose-response association. *p*-overall represents the global significance of the RCS model. *p*-linear represents the significance of the linear term. *p*-nonlinear was tested by comparing the RCS model with the linear model using a likelihood ratio test. All analyses were adjusted for age, gender, BMI Z-score, boarding status, father education level and sleep duration. Bold *p*-values indicate " <0.05".

Table S6. Summary of Restricted Cubic Spline Analyses of Associations Between Dietary Patterns, Dietary Index, and middle-to-late-stage iron deficiency (IDE and IDA).

Variable	*p*-overall	*p*-linear	*p*-nonlinear
CDGI (2021)-C score	0.117	**0.028**	0.362
Snack–fast food pattern score	**0.015**	**0.017**	**0.036**
Cereal–tuber–legume pattern score	**0.001**	**<0.001**	**0.020**
Meat–offal pattern score	**0.034**	**0.023**	0.280
Fruit–vegetable pattern score	0.395	0.969	0.365

Note: Restricted cubic spline (RCS) regression was used to evaluate the dose-response association. *p*-overall represents the global significance of the RCS model. *p*-linear represents the significance of the linear term. *p*-nonlinear was tested by comparing the RCS model with the linear model using a likelihood ratio test. All analyses were adjusted for age, gender, BMI Z-score, boarding status, moderate-to-high-intensity exercise and sleep duration. Bold *p*-values indicate " <0.05".
